# Association of *NPC1L1* and *HMGCR* gene polymorphisms with coronary artery calcification in patients with premature triple-vessel coronary disease

**DOI:** 10.1186/s12920-024-01802-0

**Published:** 2024-01-17

**Authors:** Yulong Li, Jiawen Li, Xiaofang Tang, Jingjing Xu, Ru Liu, Lin Jiang, Jian Tian, Yin Zhang, Dong Wang, Kai Sun, Bo Xu, Wei Zhao, Rutai Hui, Runlin Gao, Lei Song, Jinqing Yuan, Xueyan Zhao

**Affiliations:** National Clinical Research Center for Cardiovascular Diseases, State Key Laboratory of Cardiovascular Disease, Fuwai Hospital, National Center for Cardiovascular Diseases, Chinese Academy of Medical Sciences and Peking Union Medical College, No. 167 Beilishi Road, Xicheng District, Beijing, 100037 China

**Keywords:** NPC1L1, HMGCR, Single nucleotide polymorphisms, Coronary artery calcification, Premature triple-vessel disease

## Abstract

**Background:**

Coronary artery calcification (CAC) is a highly specific marker of atherosclerosis. Niemann-Pick C1-like 1 (NPC1L1) and 3-hydroxy-3-methylglutaryl-coenzyme A reductase (HMGCR) are the therapeutic targets of ezetimibe and statins, respectively, which are important for the progression of atherosclerosis. However, CAC’s genetic susceptibility with above targets is still unknown. We aimed to investigate the association of *NPC1L1* and *HMGCR* gene polymorphisms with CAC in patients with premature triple-vessel disease (PTVD).

**Methods:**

Four single nucleotide polymorphisms (SNPs) (rs11763759, rs4720470, rs2072183, rs2073547) of *NPC1L1*, and three SNPs (rs12916, rs2303151, rs4629571) of *HMGCR* were genotyped in 872 PTVD patients. According to the coronary angiography results, patients were divided into low-degree CAC group and high-degree CAC group.

**Results:**

A total of 872 PTVD patients (mean age, 47.71 ± 6.12; male, 72.8%) were finally included for analysis. Multivariate logistic regression analysis showed no significant association between the SNPs of *NPC1L1* and *HMGCR* genes and high-degree CAC in the total population (*P* > 0.05). Subgroup analysis by gender revealed that the variant genotype (TT/CT) of rs4720470 on *NPC1L1* gene was associated with increased risk for high-degree CAC in male patients only (OR = 1.505, 95% CI: 1.008–2.249, *P* = 0.046) in dominant model, but no significant association was found in female population, other SNPs of *NPC1L1* and *HMGCR* genes (all *P* > 0.05).

**Conclusions:**

We reported for the first time that the rs4720470 on *NPC1L1* gene was associated with high-degree CAC in male patients with PTVD. In the future, whether therapies related to this target could reduce CAC and cardiovascular events deserves further investigation.

**Supplementary Information:**

The online version contains supplementary material available at 10.1186/s12920-024-01802-0.

## Introduction

Patients with triple-vessel coronary disease (TVD) may have higher risk of death and worse cardiovascular outcomes [1], especially in those with premature coronary heart disease (CHD) due to the high likelihood for recurrence after initial events [2]. Therefore, premature triple-vessel coronary disease (PTVD) has already risen to a public health problem, which is worthy to find biomarkers for early detection and intervention. Coronary artery calcification (CAC) is a highly specific marker of atherosclerosis, and many studies have already shown that CAC is an independent predictor for long-term risk of cardiovascular events [3, 4]. It is generally believed that patients with elevated low-density lipoprotein cholesterol (LDL-C) not only have higher risk for CHD [5], but also is associated with the incidence of CAC [6], the mechanism of which may be related to its strong pathogenic effect for coronary atherosclerosis [7]. Hence, it is worth investigating whether there are common pathways between LDL-C and CAC.

The regulatory mechanisms of intracellular cholesterol could be mainly attributed as endogenous cholesterol synthesis and exogenous cholesterol uptake by extracellular LDL receptor. The former is catalyzed by 3-hydroxy-3-methylglutaryl-coenzyme A reductase (HMGCR), the target of statins, which is a rate-limiting enzyme of cholesterol synthesis [8]; the latter is regulated by Niemann-Pick C1-like 1 (NPC1L1), the target of ezetimibe, which participates in the absorption of exogenous food cholesterol [9]. A large 2 × 2 factorial mendelian randomization study [10] showed that variants of *NPC1L1* and *HMGCR* genes were related to higher risk of CHD. Moreover, our previous studies [11–13] demonstrated that single nucleotide polymorphisms (SNPs) of *NPC1L1* and *HMGCR* genes were associated with residual cholesterol risk, PTVD susceptibility, and major adverse cardiac and cerebrovascular events (MACCE) in TVD patients. However, genetic studies on the association of *HMGCR* and *NPC1L1* gene polymorphisms with CAC susceptibility has never been reported before.

Therefore, we performed this study to explore whether SNPs of *NPC1L1* and *HMGCR* genes were associated with high-degree CAC susceptibility in PTVD patients, in order to explain the mechanisms of CAC in PTVD patients from genetic perspectives, which will help to identify patients at high risk for CAC, perhaps helping to individualize the treatment of such patients in the future.

## Methods

### Study design and populations

**Fig. 1:**
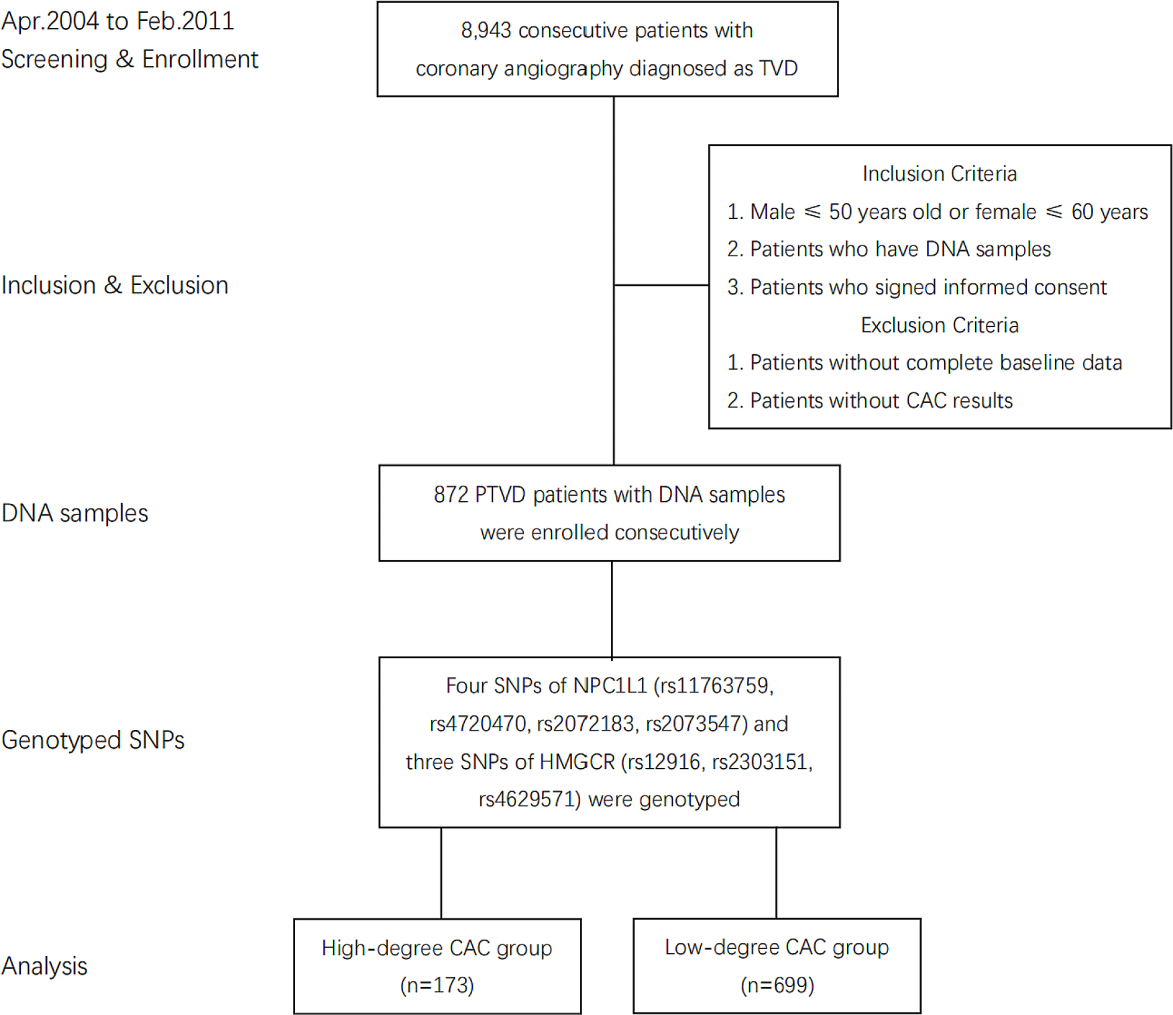
Patient flowchart. TVD: triple-vessel coronary disease; PTVD: premature triple-vessel coronary disease; SNP: single nucleotide polymorphisms; NPC1L1: Niemann-Pick C1-like 1; HMGCR: 3-hydroxy-3-methylglutaryl-coenzyme a reductase; CAC: coronary artery calcification

This was a prospective, single-center cohort study. 8,943 consecutive patients diagnosed as TVD by coronary angiography who were willing to follow-up in Fuwai Hospital were enrolled from April 2004 to February 2011. The methodology had already been described in previous studies [14, 15]. The definition of PTVD was defined as patients with TVD (angiographic stenosis of ≥ 50% in all three main coronary arteries, with or without the left main artery involved), and met the age requirement (male ≤ 50 years old or female ≤ 60 years old) [16]. Among them, a total of 1,792 patients met the PTVD criteria. Finally, 872 patients who had blood samples and met the testing criteria were enrolled in the current analysis. The precise inclusion and exclusion criteria were shown in Fig. 1. General information, baseline data, past history, and laboratory tests of all patients were collected on admission, above which were recorded in a dedicated database by independent research personnel. The Declaration of Helsinki protocols were followed. The research protocol was approved by the ethics committee of Fuwai Hospital. Written informed consent was obtained from all participants.

### Definitions and groups

Patients were subsequently categorized into low-degree CAC group or high-degree CAC group according to the calcification severity of the target lesion. Severity of calcification was classified from visual assessment via coronary angiography by two experienced interventional cardiologists who were independent of this study. None and mild calcification were defined as low-degree CAC. Moderate (radiopacities noted with cardiac motion before contrast injection) and severe calcification (radiopacities noted without cardiac motion before contrast injection) were defined as high-degree CAC [17, 18].

### Blood sampling and DNA extraction

Fasting blood samples were drawn from all patients within 24 h of admission and genomic DNA was extracted from leukocytes through the standard salting-out method [19]. The samples were stored in a refrigerator at − 80 °C. By consulting the previous literature and HapMap project (http://hapmap.ncbi.nlm.nih.gov) of the Chinese in Beijing with a minor allele frequency ≥ 0.05, the following SNPs were selected, including four *NPCIL1* sites (rs11763759, rs4720470, rs2072183, and rs2073547) and three *HMGCR* sites (rs12916, rs2303151, and rs4629571). The SNP genotyping was performed using an improved multiplex ligation detection reaction technique [20], which was newly developed by Genesky Biotechnologies Inc. (Shanghai, China) with a custom-by-design 48-Plex SNPscan™ Kit (Cat#:G0104; Genesky Biotechnologies Inc., Shanghai, China). This kit was developed according to patented SNP genotyping technology by Genesky Biotechnologies Inc., which was based on double ligation and multiplex fluorescence PCR. All probes were designed by and ordered from Genesky Biotechnologies Inc. (Shanghai, China). Our actual steps were illustrated in Supplementary Methods (Additional File 1).

### Laboratory index measurement

The blood cell counts were assayed with Sysmex XN 2000 Automatic Hemocytometer (Sysmex Corporation, Kobe, Japan). The concentrations of fasting blood glucose were measured using the glucose assay kit (Biosino Bio-Technology And Science Incorporation, Beijing, China) with glucose oxidase method. Total cholesterol with CHOD-PAP method, and triglyceride with GPO-PAP method were determined using corresponding commercially available test kits (Biosino Bio-Technology and Science Incorporation, Beijing, China). Plasma high-density lipoprotein cholesterol with chemistry modify enzyme method and LDL-C with selective melt method were determined using corresponding commercially available test kits (Minaris Medical (Shanghai) Co., Ltd., Shanghai, China). Other laboratory indicators were assayed at the biochemistry center of Fuwai Hospital by standard biochemical techniques.

### Statistical analysis

Mean with standard deviation was used to describe normally distributed continuous variables, and Student’s t-test was used to compare the differences. M (Q_1_, Q_3_) was used to describe abnormally distribute continuous variables, and Mann-Whitney U test was used to compare the differences. Frequency and percentage were used to describe categorical variables, and Pearson chi-square test or Fisher’s exact probability method were used to compare the differences. Univariate and multivariate logistic regression analysis adjusted for age and sex were used to estimate the odds ratio (OR) and 95% confidence intervals (CI) for risk of high-degree CAC associated with selected SNPs. All statistical tests were two-sided with a significant level of 0.05. Statistical analyses were performed using SPSS 26.0 software (IBM Corp., Armonk, New York, USA).

## Results

### Baseline characteristics

A total of 872 PTVD patients were finally included (mean age, 47.71 ± 6.12 years; male, 72.8%). Among them, there were 173 patients (19.8%) in the high-degree CAC group, and 699 (80.2%) patients were in the low-degree CAC group. Compared with the low-degree CAC group, patients in the high-degree CAC group were more often with prior use of statins (*P* < 0.001) and higher systolic blood pressure (*P* = 0.033) (Table 1).

**Table 1:** Baseline characteristics between two groups

Variables	High-degree CAC (*n* = 173)	Low-degree CAC (*n* = 699)	P value
Sex, M/F	122/51	513/186	0.447
Age, y	48.01 ± 6.50	47.63 ± 6.03	0.473
BMI, kg/m^2^	26.28 ± 2.75	26.49 ± 3.13	0.395
Diabetes, %	36.4	34.6	0.658
Smoking, %	55.2	54.3	0.834
Prior use of statins, %	85.5	66.7	< 0.001
Hypertension, %	62.4	61.4	0.799
SBP, mm Hg	121.88 ± 17.60	125.14 ± 18.09	0.033
DBP, mm Hg	77.30 ± 11.54	79.21 ± 11.84	0.057
TC, mmol/L	4.74 ± 1.08	4.85 ± 1.19	0.276
TG, mmol/L	2.17 ± 1.40	2.01 ± 1.08	0.146
HDL-C, mmol/L	1.02 ± 0.27	1.00 ± 0.24	0.351
LDL-C, mmol/L	2.61 ± 0.83	2.76 ± 0.97	0.061
Glucose, mmol/L	6.06 ± 2.16	6.32 ± 2.17	0.150
hs-CRP, mg/L	1.96 (0.92, 5.01)	1.90 (0.89, 4.47)	0.927

CAC: coronary artery calcification; BMI: body mass index; SBP: systolic blood pressure; DBP: diastolic blood pressure; TC: total cholesterol; TG: triglycerides; HDL-C: high density lipoprotein cholesterol; LDL-C: low density lipoprotein cholesterol; hs-CRP: high sensitive C-reactive protein

### The frequency of gene polymorphisms

The genotype distribution of polymorphisms in overall enrolled patients was conformed to Hardy–Weinberg equilibrium (both *P* > 0.05, Table S1) (Additional File 2). The frequency of gene polymorphisms of four SNPs of *NPC1L1* (rs11763759, rs4720470, rs2072183, rs2073547) and three SNPs of *HMGCR* (rs12916, rs2303151, rs4629571) were compared between high-degree CAC and low-degree CAC group, and there was no significant difference in the frequency of gene polymorphisms of above SNPs between two groups (all *P* > 0.05) (Table 2).

**Table 2:** Frequency of gene polymorphisms in two groups

Gene	SNP	Genotype	High-degree CAC (*n* = 173)	Low-degree CAC (*n* = 699)	P value
*NPC1L1*	rs11763759	TT	153 (88.4%)	622 (89.1%)	0.587
		CT	20 (11.6%)	71 (10.2%)	
		CC	0 (0.0%)	5 (0.7%)	
	rs4720470	CC	75 (43.6%)	340 (48.6%)	0.402
		CT	78 (45.3%)	278 (39.8%)	
		TT	19 (11.0%)	81 (11.6%)	
	rs2072183	GG	64 (37.0%)	278 (39.9%)	0.577
		CG	81 (46.8%)	326 (46.8%)	
		CC	28 (16.2%)	93 (13.3%)	
	rs2073547	AA	64 (37.0%)	276 (39.5%)	0.668
		GA	81 (46.8%)	327 (46.8%)	
		GG	28 (16.2%)	96 (13.7%)	
*HMGCR*	rs12916	TT	40 (23.3%)	172 (24.7%)	0.302
		TC	89 (51.7%)	317 (45.5%)	
		CC	43 (25.0%)	208 (29.8%)	
	rs2303151	CC	118 (68.2%)	440 (62.9%)	0.424
		CT	48 (27.7%)	229 (32.8%)	
		TT	7 (4.0%)	30 (4.3%)	
	rs4629571	AA	149 (86.1%)	570 (81.5%)	0.387
		AG	22 (12.7%)	120 (17.2%)	
		GG	2 (1.2%)	9 (1.3%)	

CAC: coronary artery calcification; SNP: single nucleotide polymorphisms; NPC1L1: Niemann-Pick C1-like 1; HMGCR: 3-hydroxy-3-methylglutaryl-coenzyme A reductase

### Gene polymorphisms and CAC susceptibility

#### SNPs of *NPC1L1*

After multivariate logistic regression analysis adjusted for age and sex, there was no statistical difference among rs11763759, rs4720470, rs2072183, and rs2073547 in the three genetic models between high-degree CAC group and low-degree CAC group in the total population (all *P* > 0.05).

We also conducted subgroup analysis by gender to test whether there were differences between male and female population, and the results showed that in the male population, the variant genotype (TT/CT) of rs4720470 was an independent risk factor for getting high-degree CAC in dominant model (OR: 1.505, 95% CI: 1.008–2.249, *P* = 0.046). However, the results were not the same in the female population, and there was also no statistical difference among rs11763759, rs2072183 and rs2073547 in the three genetic models between two groups (all *P* > 0.05) (Table 3).

**Table 3:** Multivariate logistic regression analysis between *NPC1L1* and *HMGCR* gene polymorphisms and coronary artery calcification

Gene	SNP	Genetic Model		Total Population (*n* = 872)			Male Population (*n* = 635)			Female Population (*n* = 237)		
				OR	95% CI	P	OR	95% CI	P	OR	95% CI	P
*NPC1L1*	rs11763759 T > C	Dominant	CC + CT/TT	1.069	0.633–1.804	0.803	0.967	0.510–1.832	0.918	1.331	0.527–3.365	0.545
		Recessive	CC/CT + TT	–	–	–	–	–	–	–	–	–
		Codominant	CC/CT/TT	0.997	0.608–1.635	0.991	0.893	0.493–1.617	0.708	1.331	0.527–3.365	0.545
	rs4720470 C > T	Dominant	TT + CT/CC	1.224	0.874–1.713	0.239	1.505	1.008–2.249	0.046	0.742	0.396–1.391	0.352
		Recessive	TT/CT + CC	0.945	0.556–1.607	0.836	1.281	0.705–2.328	0.416	0.397	0.115–1.373	0.144
		Codominant	TT/CT/CC	1.100	0.862–1.404	0.442	1.311	0.985–1.746	0.063	0.710	0.439–1.150	0.164
	rs2072183 G > C	Dominant	CC + GC/GG	1.126	0.799–1.589	0.498	1.079	0.719–1.619	0.712	1.266	0.657–2.440	0.481
		Recessive	CC/GC + GG	1.255	0.792–1.990	0.334	1.227	0.714–2.108	0.460	1.249	0.515–3.031	0.623
		Codominant	CC/GC/GG	1.128	0.885–1.436	0.330	1.097	0.825–1.458	0.524	1.196	0.753-1.900	0.449
	rs2073547 A > G	Dominant	GG + GA/AA	1.107	0.785–1.562	0.563	1.029	0.687–1.542	0.889	1.343	0.691–2.609	0.385
		Recessive	GG/GA + AA	1.216	0.768–1.924	0.405	1.171	0.683–2.007	0.567	1.260	0.519–3.056	0.609
		Codominant	GG/GA/AA	1.108	0.871–1.411	0.404	1.058	0.797–1.404	0.696	1.235	0.775–1.968	0.374
*HMGCR*	rs12916 T > C	Dominant	CC + CT/TT	1.072	0.722–1.591	0.732	1.391	0.865–2.238	0.174	0.539	0.259–1.122	0.099
		Recessive	CC/CT + TT	0.772	0.526–1.132	0.186	0.798	0.501–1.270	0.341	0.710	0.361–1.396	0.321
		Codominant	CC/CT/TT	0.929	0.738–1.170	0.531	1.035	0.788–1.359	0.804	0.708	0.460–1.092	0.118
	rs2303151 C > T	Dominant	TT + CT/CC	0.789	0.553–1.125	0.190	0.728	0.474–1.117	0.146	0.945	0.497–1.798	0.863
		Recessive	TT/CT + CC	0.927	0.400-2.151	0.860	0.386	0.089–1.668	0.202	2.179	0.687–6.916	0.186
		Codominant	TT/CT/CC	0.836	0.617–1.131	0.245	0.721	0.493–1.055	0.092	1.105	0.665–1.838	0.699
	rs4629571 A > G	Dominant	GG + GA/AA	0.707	0.441–1.134	0.150	0.657	0.365–1.181	0.160	0.835	0.373–1.871	0.661
		Recessive	GG/GA + AA	0.869	0.186–4.068	0.858	2.182	0.394–12.08	0.371	–	–	–
		Codominant	GG/GA/AA	0.745	0.485–1.145	0.179	0.746	0.438–1.271	0.281	0.757	0.365–1.569	0.454

OR: Odds Ratio; CI: Confidence Interval; SNP: Single nucleotide polymorphism; NPC1L1: Niemann-Pick C1-like 1; HMGCR: 3-hydroxy-3-methylglutaryl-coenzyme A Reductase

#### SNPs of *HMGCR*

After multivariate logistic regression analysis adjusted for age and sex, there was no statistical difference among rs12916, rs2303151, and rs4629571 in the three genetic models between high-degree CAC group and low-degree CAC group in the total population (all *P* > 0.05). Even in subgroup analysis by gender, the results were also not significant in both the male and the female population (all *P* > 0.05) (Table 3).

### Sensitivity analysis

In this study, the above results could be affected by the prior use of statins. Therefore, we conducted a sensitivity analysis in patients without prior use of statins (*n* = 258) to test whether the above results were still significant. In the male population without prior use of statins, the variant genotype (TT/CT) of rs4720470 on *NPC1L1* gene was still an independent risk factor for getting high-degree CAC in dominant model (OR: 3.029, 95% CI: 1.102–8.327, *P* = 0.032) and in codominant model (OR: 2.274, 95% CI: 1.162–4.450, *P* = 0.016). We also found that in the total population without prior use of statins, the variant genotype (TT/CT) of rs4720470 was an independent risk factor for getting high-degree CAC in codominant model (OR: 1.865, 95% CI: 1.040–3.345, *P* = 0.037).

Nevertheless, there was still no significant result in the female population without prior use of statins, as well as the SNPs of *HMGCR* (all *P* ≥ 0.05), but interestingly, we found that the correlation between the variant genotype (CC/CT) of rs12916 on *HMGCR* gene and high-degree CAC was almost close to statistical significance in the total population without prior use of statins (*P* = 0.05). Above results of the sensitivity analysis were almost consistent with our primary results, suggesting the stability of our investigation (Table 4).

**Table 4:** Sensitivity analysis in patients without prior use of statins

Gene	SNP	Genetic Model		Total Population (*n* = 258)			Male Population (*n* = 175)			Female Population (*n* = 83)		
				OR	95% CI	P	OR	95% CI	P	OR	95% CI	P
*NPC1L1*	rs11763759 T > C	Dominant	CC + CT/TT	1.215	0.387–3.810	0.739	1.474	0.448–4.850	0.523	–	–	–
		Recessive	CC/CT + TT	–	–	–	–	–	–	–	–	–
		Codominant	CC/CT/TT	1.151	0.384–3.448	0.802	1.360	0.440–4.202	0.593	–	–	–
	rs4720470 C > T	Dominant	TT + CT/CC	2.373	0.972–5.796	0.058	3.029	1.102–8.327	0.032	0.950	0.149–6.068	0.957
		Recessive	TT/CT + CC	2.261	0.766–6.676	0.140	3.029	0.864–10.62	0.083	1.483	0.142–15.51	0.742
		Codominant	TT/CT/CC	1.865	1.040–3.345	0.037	2.274	1.162–4.450	0.016	1.091	0.295–4.037	0.896
	rs2072183 G > C	Dominant	CC + GC/GG	0.898	0.384–2.101	0.804	0.880	0.338–2.295	0.794	0.873	0.136–5.614	0.887
		Recessive	CC/GC + GG	2.115	0.832–5.586	0.114	1.760	0.583–5.308	0.316	3.561	0.507–25.01	0.202
		Codominant	CC/GC/GG	1.221	0.685–2.177	0.499	1.127	0.587–2.167	0.719	1.488	0.420–5.274	0.538
	rs2073547 A > G	Dominant	GG + GA/AA	0.860	0.366–2.023	0.730	0.824	0.313–2.168	0.695	0.833	0.130–5.349	0.848
		Recessive	GG/GA + AA	2.004	0.775–5.180	0.152	1.619	0.539–4.862	0.391	3.561	0.507–25.01	0.202
		Codominant	GG/GA/AA	1.173	0.660–2.087	0.587	1.071	0.558–2.054	0.838	1.464	0.409–5.235	0.558
*HMGCR*	rs12916 T > C	Dominant	CC + CT/TT	7.639	0.999–58.43	0.050	6.555	0.844–50.90	0.072	–	–	–
		Recessive	CC/CT + TT	1.550	0.643–3.737	0.329	1.222	0.434–3.439	0.704	2.854	0.437–18.62	0.273
		Codominant	CC/CT/TT	1.857	0.987–3.496	0.055	1.695	0.830–3.314	0.152	2.840	0.542–14.89	0.217
	rs2303151 C > T	Dominant	TT + CT/CC	1.603	0.697–3.686	0.267	1.130	0.434–2.941	0.802	6.610	0.700-62.42	0.099
		Recessive	TT/CT + CC	1.639	0.341–7.873	0.537	0.868	0.101–7.472	0.898	3.901	0.333–45.72	0.278
		Codominant	TT/CT/CC	1.458	0.763–2.788	0.254	1.063	0.490–2.307	0.878	3.392	0.896–12.83	0.072
	rs4629571 A > G	Dominant	GG + GA/AA	0.998	0.322–3.092	0.998	1.027	0.277–3.804	0.968	1.033	0.106–10.01	0.978
		Recessive	GG/GA + AA	–	–	–	–	–	–	–	–	–
		Codominant	GG/GA/AA	0.910	0.322–2.570	0.859	0.986	0.279–3.483	0.983	0.834	0.128–5.441	0.849

OR: odds ratio; CI: confidence interval; SNP: single nucleotide polymorphism; NPC1L1: Niemann-Pick C1-like 1; HMGCR: 3-hydroxy-3-methylglutaryl-coenzyme a reductase

## Discussion

We genotyped four SNPs of *NPC1L1* (rs11763759, rs4720470, rs2072183, rs2073547) and three SNPs of *HMGCR* (rs12916, rs2303151, rs4629571) in 872 PTVD patients. The results are as follows: (1) We reported for the first time that the variant genotype (TT/CT) of rs4720470 on *NPC1L1* gene was associated with increased risk for high-degree CAC in male patients with PTVD in dominant model, but no significant result was found in female population; (2) We did not find any relationship between these SNPs (rs12916, rs2303151, rs4629571) of *HMGCR* and high-degree CAC, no matter in male or female population.

To our knowledge, this is the first report that the variant genotype (TT/CT) of rs4720470 on *NPC1L1* gene causes an increased risk of high-degree CAC in male patients with PTVD. We have not found any literature on the association of *NPC1L1* gene polymorphisms with CAC susceptibility, and the exact mechanism by which this mutation leads to CAC is unclear. The possible reason for this result is that mutations in this locus may affect lipid metabolism, which in turn affects the progression of coronary atherosclerosis and CAC. Some studies supported that mutations on *NPC1L1* gene leaded to its loss of function. Cohen et al. [21] had once demonstrated that multiple rare variations in *NPC1L1* gene were associated with decreased cholesterol absorption and low level of plasma LDL-C. Meanwhile, other researches [22, 23] also reported that mutations on *NPC1L1* gene were found to be associated with a reduced risk of CHD, with a corresponding reduction in LDL-C. However, other studies supported that mutations on *NPC1L1* gene leaded to its gain of function. Polisencki et al. [24] indicated that variations on the *NPC1L1* gene were associated with higher total and LDL cholesterol levels and increased risk of CHD. Muendlein et al. [25] also reported that variations of *NPC1L1* gene, particularly the SNP of rs55837134, showed a predictive impact on cardiovascular events. At the same time, our previous studies [11, 12] have already demonstrated that the variant genotype of rs4720470 on *NPC1L1* gene was associated with PTVD susceptibility and MACCE in TVD patients. Our current study founded that variant genotype of rs4720470 on *NPC1L1* gene could increase CAC in PTVD patients. We speculated that the variation of this SNP might activate *NPC1L1* gene function, increasing the level of LDL-C, and then leads to the progression of atherosclerosis and CAC. However, few studies have been reported on whether inhibitors of NPC1L1, like ezetimibe, could reduce CAC. An animal study [26] demonstrated that, compared with statins alone, the combination of ezetimibe significantly reduced the degree of vascular calcification in ApoE^−/−^ and CHOP^−/−^ mice. Another animal study [27] also incurred the inhibitory effect of ezetimibe on the development of lipid-rich plaque, the mechanisms of which may be related to the improved endothelial dysfunction, suppressed oxidative stress and the ubiquitination-proteasome system. Both of these two studies suggest a protective effect of ezetimibe on atherosclerosis and vascular calcification. In a clinical study, Hougaard et al. [28] founded that statins monotherapy significantly increased CAC in patients with ST-elevation myocardial infarction, but not in patients treated with statins combined with ezetimibe. Therefore, we speculated that inhibition of NPC1L1 might reduce CAC. In the future, further studies related to its molecular mechanisms could be carried out to investigate its potential pathogenesis.

However, our study found that the variant genotype (TT/CT) of rs4720470 on *NPC1L1* gene was associated with high-degree CAC only in the male PTVD population, but not in the female PTVD population. The reasons for this result may be described as follows. Firstly, our study was carried out in the PTVD population, among which the female age was ≤ 60 years old and was relatively young, so the coronary artery in this population may not have developed to a high degree of calcification. Secondly, since previous studies showed that atherosclerosis differed by gender [29] and estrogen had protective impact on the progression of atherosclerosis [30, 31]. Several previous studies had already confirmed the protective mechanisms of estrogen on vascular calcification, including ERα-mediated Gas6 transactivation [32], upregulating BMP2 signaling pathway [33], and changing vascular RANKL system [34]. The women in our study were under 60 years old, and the coronary arteries in these population may not have developed to high-degree CAC due to the effect of estrogen and other protective factors. Thirdly, our study was conducted in the Chinese population, and previous study [35] have confirmed that the degree of CAC in Chinese is relatively low (77% that of whites), and this could be one of the reasons for our results. A study from MSEA cohort [36] indicated that the degree of CAC was correlated with age, gender and race, that is, male, elderly and whites were more likely to find high-degree CAC, which further supports why we couldn’t find significant result in premature female population. Lastly, it was worth emphasizing that there was a significant disproportion between the number of men and women, which might affect the results of the analyses. In the future, whether this locus mutation is associated with CAC in women older than 60 years of age deserves further investigation.

Meanwhile, we also did not find any significant correlation between these SNPs of *HMGCR* and high-degree CAC, no matter in male or female population. In recent years, several clinical and genetic studies have conducted on the association of *HMGCR* gene polymorphisms with lipid levels or CHD risk. Several studies [37–39] have indicated that presence of *HMGCR* mutations was correlated with affected statins therapy and LDL-C level, which is consistent with our current published research [13] that the variant genotype of rs12916 within *HMGCR* gene may incur a significantly higher risk of residual cholesterol risk in PTVD patients treated with statins. As for risk of CHD, study conducted by Ference et al. [10] suggested that polymorphisms of *HMGCR* gene were related to lower risk of CHD. Kettunen et al. [40] and our previous study [11, 12] reported that the reduced expression of rs12916 on *HMGCR* gene could reduce the risk of CHD. As a result, we speculated mutations on *HMGCR* gene might change its function and in turn, change in lipid level and progression of atherosclerosis. However, we did not find any relationship between these SNPs of *HMGCR* and CAC. In review of previous SNP studies on *HMGCR* gene, we did not retrieve any report on its relationship to CAC. In recent years, the management of CHD patients is becoming more standardized, and statins have been widely used in these patients. Statins increased the risk of CAC by potentially making plaque’s microcalcifications more fused and denser, and then increasing its steadily [41]. Previous studies [42, 43] have already demonstrated that statins pharmacotherapy increased CAC and 70.4% of our study population had prior use of statins, so this could be the potential reason why we couldn’t find significant correlation between SNPs of *HMGCR* gene and CAC. Interestingly, in our sensitivity analysis for patients without prior use of statins, the correlation between the variant genotype (CC/CT) of rs12916 on *HMGCR* gene and high-degree CAC was close to statistical significance in dominant model (*P* = 0.05). In the future, larger scale of investigation could be carried out to further test their relationship, as well as functional studies to explore potential mechanisms.

Although the genetic factors involved in CHD have been confirmed by genome-wide association studies, substantial association between gene polymorphisms and the occurrence and prognosis of CHD remains to be clarified [44]. To our knowledge, we first reported the relationship between lipid regulatory genes *NPC1L1* and *HMGCR* and CAC, revealing that variations on lipid regulatory genes play a crucial role in the occurrence and progression of atherosclerosis. The findings of these relationships might help us to predict high risk of CAC in patients with PTVD, and seek for new therapeutic targets, aiming to reduce CAC and further improve their cardiovascular outcomes. In the future, whether editing of these genes or inhibition of their proteins’ function can reduce CAC and improve cardiovascular outcomes deserves further exploration. Meanwhile, except for *NPC1L1* and *HMGCR* genes, other lipid regulatory genes like *PCSK9*, *APOB*, *ABCG5-G8*, *KCNK5*, *LDLR*, *LPA*, et al. have also been provided the evidence to their critical role in CHD risk [45], and further investigations on these genes’ SNPs with CAC susceptibility is worthy.

### Limitations

There are some limitations to our study that should be taken into consideration. First, this is a single-center cohort study, which may limit its generalizability. Second, even though a large scale of population had been screened, the final sample size was relatively small, which required a larger size of sample to validate our conclusions. Third, lack of the gynecological disease history and estrogen level data of female patients, and the disproportion between the number of men and women may further affect the final results. Fourth, our study only enrolled Chinese population, so whether our conclusions differ from different races needs further investigation in the future.

## Conclusions

We report variant genotype (TT/CT) of rs4720470 on *NPC1L1* gene is related to the risk of high-degree CAC in male patients with PTVD, which help clinicians to early identify high-risk patients, and suggests that *NPC1L1* may act as an important channel for CAC from the perspective of genetic polymorphisms. However, we do not find the correlation between these SNPs of *NPC1L1* and high-degree CAC in female population with PTVD. The relationship between these SNPs of *HMGCR* and high-degree CAC is not significant in both male and female population with PTVD.

### Electronic supplementary material

Below is the link to the electronic supplementary material.

Supplementary Material 1

Supplementary Material 2

## Data Availability

Due to ethical restrictions related to the consent given by subjects at the time of study commencement, our datasets are available from the corresponding author upon reasonable request after permission of the Institutional Review Board of National Clinical Research Center for Cardiovascular Diseases, State Key Laboratory of Cardiovascular Disease, Fuwai Hospital, National Center for Cardiovascular Diseases.

## Abbreviations

TVD: triple-vessel coronary disease
PTVD: premature triple-vessel coronary disease
CHD: coronary heart disease
CAC: coronary artery calcification
LDL-C: low-density lipoprotein cholesterol
HMGCR: 3-hydroxy-3-methylglutaryl-coenzyme A reductase
NPC1L1: Niemann-Pick C1-like 1
SNPs: single nucleotide polymorphisms
MACCE: major adverse cardiac and cerebrovascular disease
OR: odds ratio
CI: confidence interval
