# Supplementary material for: Association of NPC1L1 and HMGCR gene polymorphisms with coronary artery calcification in patients with premature triple-vessel coronary disease
Source: BMC Med Genomics. 2024 Jan 17;17:22. doi: 10.1186/s12920-024-01802-0 (PMC10795340; doi:10.1186/s12920-024-01802-0)
Supplement: Supplementary file 1 — Supplementary Material 1 [file 12920_2024_1802_MOESM1_ESM.doc]

**Supplementary Methods.** Actual steps for DNA extraction

Briefly, 100–200 ng of DNA sample was first denatured at 98 ℃ for 5 min in a 10 μL reaction containing 2.5 μL 4x DNA lysis buffer and then mixed well with a 10 μL ligation premix composed of 2 μL 10x ligase buffer, 0.5 μL ligase, 1 mL probe mix, and 7.5 μL Milli-Q water. The ligation reaction was carried out in an ABI2720 thermal cycler. The ligation cycling program was 4 cycles x (94℃ 1 min, 58℃ 4 h); 94℃ 2 min; hold at 72℃. Two 48-plex fluorescence PCR reactions were performed for each ligation product. PCR reactions were prepared in a 20 μL mixture containing 2x PCR master mix, 1 μL primer mix set A or set B, and 1 μL ligation product. The PCR cycling conditions were as follows: 95℃ for 2 min; followed by 9 cycles of 94℃ for 20 s, 65-0.5℃/cycle for 40 s, and 72℃ 1 min 30 s; then 25 cycles of 94℃ for 20 s, 57℃ for 40s, and 72℃ 1 min 30 s; and a final extension of 68℃ for 60 min and holding at 4℃. PCR products were separated and detected by capillary electrophoresis in an ABI3730XL sequencer. Raw data were analyzed according to the information obtained for the labeling dye color and fragment size of the allele-specific ligation-PCR product. Genotyping was conducted without any knowledge regarding the subject’s case or control status. For quality control, repeated analyses were accomplished to guarantee the genotyping quality by randomly choosing 4 % of samples with high DNA quality.
