# Supplementary material for: Association of NPC1L1 and HMGCR gene polymorphisms with coronary artery calcification in patients with premature triple-vessel coronary disease
Source: BMC Med Genomics. 2024 Jan 17;17:22. doi: 10.1186/s12920-024-01802-0 (PMC10795340; doi:10.1186/s12920-024-01802-0)
Supplement: Supplementary file 2 — Supplementary Material 2 [file 12920_2024_1802_MOESM2_ESM.doc]

**Table S1.** Hardy–Weinberg equilibrium of genotype of *NPC1L1* and *HMGCR* genes

| **Gene** | **SNPs** | **Major allele** | **Minor allele** | **Frequency of Genotype** | **Homozygote for the major allele (%)** | **Heterozygote (%)** | **Homozygote for the minor allele** | **HWE-*P* value** |
| --- | --- | --- | --- | --- | --- | --- | --- | --- |
| *NPC1L1* | rs11763759 | *T* | *C* |  | *TT* | *CT* | *CC* |  |
|  |  | 1643/1744 (94.2%) | 101/1744 (5.8%) | Observed value | 776 (89.0%) | 91 (10.4%) | 5 (0.6%) | 1.000* |
|  |  |  |  | Expected value | 774 (88.8%) | 95 (10.9%) | 3 (0.3%) |  |
|  | rs4720470 | *C* | *T* |  | *CC* | *CT* | *TT* |  |
|  |  | 1188/1744 (68.1%) | 556/1744 (31.9%) | Observed value | 416 (47.7%) | 356 (40.8%) | 100 (11.5%) | 0.994 |
|  |  |  |  | Expected value | 404 (46.4%) | 379 (43.4%) | 89 (10.2%) |  |
|  | rs2072183 | *G* | *C* |  | *GG* | *CG* | *CC* |  |
|  |  | 1095/1744 (62.8%) | 649/1744 (37.2%) | Observed value | 344 (39.4%) | 407 (46.7%) | 121 (13.9%) | 0.972 |
|  |  |  |  | Expected value | 344 (39.4%) | 407 (46.7%) | 121 (13.9%) |  |
|  | rs2073547 | *A* | *G* |  | *AA* | *GA* | *GG* |  |
|  |  | 1088/1744 (62.4%) | 656/1744 (37.6%) | Observed value | 340 (39.0%) | 408 (46.8%) | 124 (14.2%) | 1.000 |
|  |  |  |  | Expected value | 340 (39.0%) | 409 (46.9%) | 123 (14.1%) |  |
| *HMGCR* | rs12916 | *T* | *C* |  | *TT* | *CT* | *CC* |  |
|  |  | 836/1744 (47.9%) | 908/1744 (52.1%) | Observed value | 215 (24.6%) | 406 (46.6%) | 251 (28.8%) | 0.960 |
|  |  |  |  | Expected value | 200 (22.9%) | 436 (50.0%) | 236 (27.1%) |  |
|  | rs2303151 | *C* | *T* |  | *CC* | *CT* | *TT* |  |
|  |  | 1393/1744 (79.9%) | 351/1744 (20.1%) | Observed value | 558 (64.0%) | 277 (31.8%) | 37 (4.2%) | 0.980 |
|  |  |  |  | Expected value | 557 (63.9%) | 280 (32.1) | 35 (4.0%) |  |
|  | rs4629571 | *A* | *G* |  | *AA* | *GA* | *GG* |  |
|  |  | 1580/1744 (90.6%) | 164/1744 (9.4%) | Observed value | 719 (82.5%) | 142 (16.3%) | 11 (1.2%) | 0.909 |
|  |  |  |  | Expected value | 716 (82.1) | 148 (17.0%) | 8 (0.9%) |  |

***Note:*** *HMGCR, 3-hydroxy-3-methylglutaryl-coenzyme A reductase; NPC1L1, Niemann-Pick C1-like 1;*

*Compared by Fisher’s exact test.
